# Functional Analysis of *OsDRP2B* in Rice Root Development

**DOI:** 10.3390/plants15020313

**Published:** 2026-01-21

**Authors:** Lihuiying Jia, Zhiqiang Guo, Yunyan Hua, Qi Zhu, Fengyi Zhou, Qiuping Li, Xu Li, Mengsha Li, Mengting Wang, Yujie Chen, Xiaofei Wang, Tao Ma, Wona Ding

**Affiliations:** 1 Ningbo Key Laboratory of Agricultural Germplasm Resources Mining and Environmental Regulation, College of Science and Technology, Ningbo University, Ningbo 315000, China; 2 Cixi Modern Agricultural Development Zone Management Center, Ningbo 315000, China; 3 State Key Laboratory of Subtropical Silviculture, Zhejiang A&F University, Hangzhou 310000, China; 4 Chongqing Research Institute, Shanghai Jiao Tong University, Chongqing 404100, China

**Keywords:** *OsDRP2B*, Dynamin proteins, *Oryza sativa*, ROS, root growth

## Abstract

Rice (*Oryza sativa* L.) root system plays a critical role in water and nutrient uptake, influencing overall plant growth and crop yield. In this study, we characterized the *Osdrp2b* mutant, which exhibits a short-root phenotype and was identified through map-based cloning. The *Osdrp2b* mutation was traced to the gene encoding a dynamin-related protein, and the mutant displayed reduced cell elongation and impaired cell division in the root tip. Further analysis revealed that ROS (reactive oxygen species) accumulation was elevated in the mutant roots, and treatment with ROS inhibitors restored root elongation in the *Osdrp2b* mutant, indicating that altered ROS homeostasis is associated with the phenotype. Transcriptomic analysis highlighted the differential expression of genes involved in cell wall organization and hydrogen peroxide catabolism. Agronomic evaluations of the *Osdrp2b* mutant demonstrated compromised shoot growth, reduced tiller number, and lower seed setting rates, indicating the impact of the mutation on rice yield. Overall, these results suggest that *OsDRP2B* is involved in regulating root growth, potentially through effects on ROS homeostasis and associated signaling networks. These findings provide a basis for future studies on improving rice root development and agronomic performance.

## 1. Introduction

Rice (*Oryza sativa* L.), one of the most important staple crops, supports over half the global population [1]. Economic models predict that by 2050, crop yields will be insufficient to meet the demands of the growing global population, emphasizing the urgent need to improve grain production [2]. The root system is a critical organ responsible for water and nutrient uptake, as well as providing anchorage. Serving as the primary interface between plants and the soil, root system architecture is closely linked to shoot morphology and crop yield [3]. Therefore, breeding strategies targeting root traits have garnered considerable attention from researchers. As a model monocot plant, rice has a typical fibrous root system comprising seminal roots, crown roots, lateral roots, and root hairs [4]. Numerous studies have demonstrated the indispensable role of the rice root system in yield formation [5,6]. Given the crucial role of rice roots in yield formation, understanding the molecular mechanisms that govern root development is essential for improving rice production.

Reactive oxygen species (ROS), including free radical species such as superoxide (O_2_^−^) and non-radical species like hydrogen peroxide (H_2_O_2_), are now recognized as crucial signaling molecules involved in various developmental processes, including root development [7]. Appropriate ROS distribution and homeostasis are required for maintaining root meristem activity, root elongation, and differentiation [8,9,10,11]. The regulatory function of ROS requires finely tuned homeostasis, maintained by a balance between ROS production and scavenging. In *Arabidopsis thaliana*, a highly dynamic and complementary network of over 150 genes has been identified as being involved in ROS homeostasis, some of which are also implicated in root development [12]. The protein phosphatase LIKE SEX FOUR2 (LSF2), identified as a key regulator of ROS homeostasis under oxidative stress, influenced root development [13]. Several studies in rice have also revealed the role of ROS homeostasis in root development. For example, altered ROS levels caused by overexpression or loss of function of specific genes result in defective root elongation and development [14,15]. Despite these advances, the precise mechanisms governing ROS homeostasis regulation in rice roots remain largely unclear.

Dynamin-related proteins (DRPs) are large multidomain GTPases that mediate membrane remodeling events essential for intracellular trafficking. In animal cells, dynamins assemble into helical structures at the necks of budding vesicles, facilitating vesicle scission via GTP hydrolysis [16]. In plants, two major DRP subfamilies-DRP1 and DRP2-have been identified and shown to function in various post-Golgi trafficking pathways, including clathrin-mediated endocytosis and cell plate formation. In *A. thaliana*, DRP family members have been shown to play critical roles in plant cytokinesis, polar cell expansion, and vacuolar trafficking [17,18,19]. In rice, DRPs have been implicated in cell wall biosynthesis, plant height regulation, and plant immunity [20,21,22]. A DRP2 family member was shown to participate in membrane trafficking related to cell wall biosynthesis, as mutants exhibited altered mechanical strength and secondary wall structure [20]. Additionally, DRPs also regulate lateral root development. The *drp* mutants exhibited increased LR number and diameter, which are associated with reduced endocytic activity and altered auxin signaling [23]. Despite these advances, the function of DRPs in regulating root growth in rice remains poorly characterized. Notably, although both membrane trafficking and ROS homeostasis are essential for root development, whether DRP family proteins are involved in the regulation of ROS homeostasis during rice root growth has not yet been reported. In the present study, a rice mutant exhibiting a short root phenotype was identified and named *Osdrp2b* through map-based cloning. Treatment of the *Osdrp2b* mutant with a ROS inhibitor partially restored the root phenotype to wild-type levels, suggesting that altered ROS homeostasis is associated with the mutant phenotype. Our findings indicate that *OsDRP2B* may regulate rice root development, potentially through effects on ROS homeostasis.

## 2. Results

### 2.1. Characterization of Root Growth Defects in the Osdrp2b Mutant

**Figure 1:**
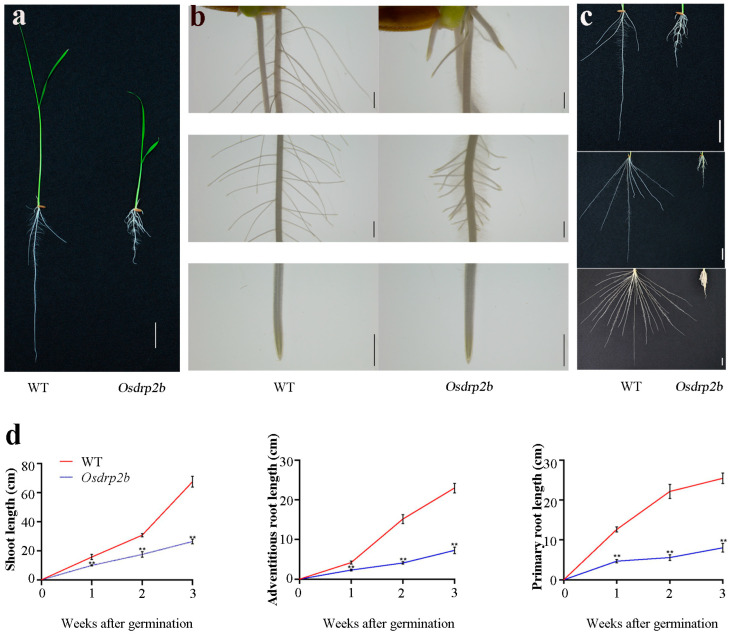
Phenotypic characterization of the *Osdrp2b* mutant. (**a**) Seedling phenotypes of WT and *Osdrp2b* grown in rice solution culture for 7 days. bar = 2 cm. (**b**) Stereomicroscope images of WT and *Osdrp2b* roots. bars = 100 μm. (**c**) Root phenotype of WT and *Osdrp2b* from 1 to 3 weeks after germination. bars = 2 cm. (**d**) Quantification of shoot length, adventitious root length, and primary root length of WT and *Osdrp2b* from 1 to 3 weeks after germination. Values are means ± SE. Significant differences were determined using Student’s *t*-test, and asterisks (**) indicating a significant difference (*p* < 0.01) from WT.

To investigate genes involved in rice root development, we screened a mutant library of *Oryza sativa* L. *indica* cv. Kasalath generated through EMS (ethylmethane sulfonate) mutagenesis. Among these, a mutant with a severe short-root phenotype attracted our attention. Subsequent map-based cloning identified the causative mutation as *Osdrp2b*. As shown in Figure 1a, both primary and adventitious root lengths were markedly reduced in the *Osdrp2b* mutant compared to the wild type (WT). Moreover, shoot growth was also compromised. Stereomicroscopic analysis further confirmed the short-root phenotype of the *Osdrp2b* mutant compared with the WT (Figure 1b). A time-course analysis was subsequently conducted to confirm these observations. As shown in Figure 1c,d, the primary root, adventitious roots, and shoot of the *Osdrp2b* mutant exhibited severe growth retardation compared to the WT. Collectively, these results suggest that the *Osdrp2b* mutation impairs root development in rice.

**Figure 2:**
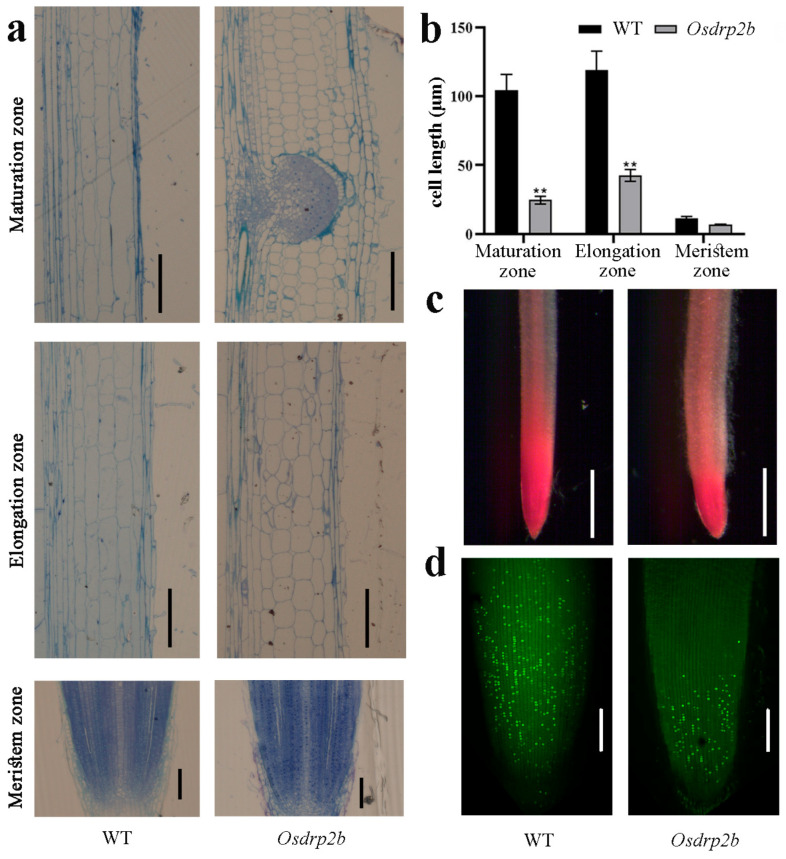
Root cell division and elongation analysis in the *Osdrp2b* mutant. (**a**) Longitudinal sections of the maturation zone (top), elongation zone (middle), and meristem zone (bottom) in 3-day-old WT and *Osdrp2b* roots. bars = 100 μm. (**b**) Quantification of cell length in roots of 3-day-old WT and *Osdrp2b* plants. Values are means ± SE. Statistical significance was determined using Student’s *t*-test, and asterisks (**) indicate a significant difference from WT (*p* < 0.01). (**c**) Acetocarmine staining of root tips in WT and *Osdrp2b*. Scale bars = 500 μm. (**d**) EdU staining of 4-day-old WT and *Osdrp2b* root tips to visualize S-phase entry. Scale bars = 100 μm.

To elucidate the cellular basis of the short-root phenotype in *Osdrp2b*, we performed a root longitudinal section analysis. As shown in Figure 2a,b, the average cell length in the elongation and maturation zones was significantly reduced in the *Osdrp2b* mutant compared to the WT. This observation indicates that cell elongation is compromised in the root tips of *Osdrp2b* mutants. To explore potential changes in radial growth, we also conducted a root cross-section analysis. As shown in Supplementary Figure S1a, *Osdrp2b* roots exhibited an increased diameter in both the elongation and maturation zones compared to the WT. Quantitative analysis further revealed that the width of cortical cells in these zones was significantly greater in *Osdrp2b* than in the WT (Supplementary Figure S1b), indicating that *Osdrp2b* roots undergo altered radial expansion of root cells.

Meristematic activity in root tips was further evaluated using acetocarmine and EdU staining. As shown in Figure 2c, acetocarmine staining demonstrated a markedly smaller stained region in the mutant compared to WT, indicating reduced cell division activity. Consistently, EdU incorporation was substantially lower in *Osdrp2b* than in the WT (Figure 2d). These findings suggest that the short-root phenotype in *Osdrp2b* mutants may result from defects in both cell elongation and cell division activity at the root tip, accompanied by abnormal radial cell expansion.

### 2.2. Map-Based Cloning of the OsDRP2B

The causal gene responsible for the root development defect in *Osdrp2b* was identified through a map-based cloning approach. Crossing the *Osdrp2b* mutant with the *japonica* rice variety Nipponbare produced F_1_ progeny exhibiting wild-type phenotypes, indicating that the mutation is recessive. Self-pollination of the F_1_ plants resulted in an F_2_ population with phenotypic segregation: 1412 individuals exhibited normal root development, while 519 individuals displayed the short-root phenotype. Chi-square analysis (*χ*^2^ = 3.53, *p* > 0.05) confirmed a 3:1 segregation ratio. These results suggest that the *Osdrp2b* phenotype is controlled by a single recessive gene.

**Figure 3:**
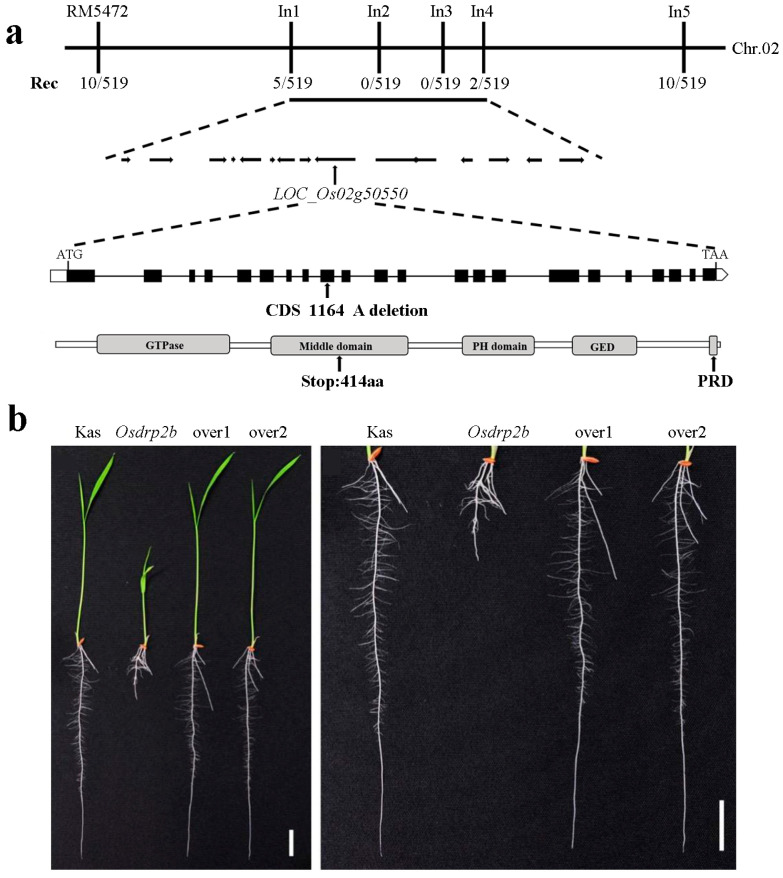
Map-based cloning of *OsDRP2B*. (**a**) The *OsDRP2B* locus was mapped to chromosome 2 between the markers In1 and In4. Rec represents recombination frequency. Black boxes and lines indicate exons and introns, respectively. The arrow marks the adenine deletion. (**b**) Complementation analysis of *Osdrp2b*. Two independent lines of *OsDRP2B*-overexpressing transgenic plants (over1 and over2) in the *Osdrp2b* mutant background are shown. Scale bars = 2 cm.

For preliminary mapping, we employed 98 pairs of SSR markers distributed across the 12 rice chromosomes and identified a linkage with the molecular marker RM5472 on chromosome 2. To further refine the mapping, we expanded the population size and developed InDel-specific markers, which localized the gene to a 109 kb region between markers In1 and In4 (Figure 3a). This region contains 15 candidate genes, as annotated by the National Rice Data Center. Sequencing of these candidate genes revealed that the causative mutation lies within the *OsDRP2B* (*LOC_Os02g50550*). *OsDRP2B* encodes a dynamin-related protein consisting of 923 amino acids. The *Osdrp2b* mutation results from a deletion of an adenine (A) at nucleotide position 1164 in the coding sequence, causing a premature stop codon and truncation of the protein at amino acid 414 (Figure 3a).

To confirm that the short-root phenotype was caused by the *OsDRP2B* mutation, we performed a transgenic complementation assay. Transgenic plants carrying *35S::CDS-OsDRP2B* exhibited root lengths comparable to those of the wild-type plants (Figure 3b). These results demonstrate that the short-root phenotype in *Osdrp2b* is attributable to the mutation in the *OsDRP2B*.

**Figure 4:**
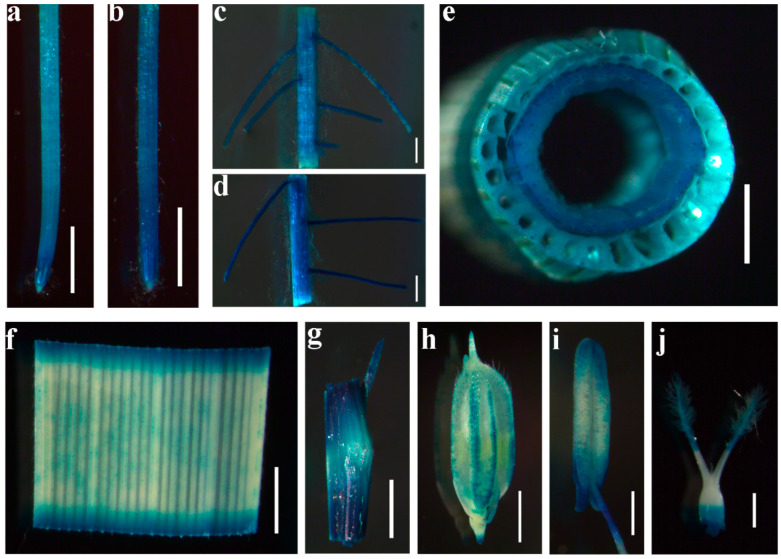
Expression pattern of *OsDRP2B*. Expression pattern of *OsDRP2B* in transgenic rice lines expressing a GUS reporter driven by the *OsDRP2B* promoter. Histochemical GUS staining revealed expression in (**a**) primary root tip, (**b**) adventitious root tip, (**c**) maturation zone of the primary root, (**d**) maturation zone of the adventitious root, (**e**) stem, (**f**) leaves, (**g**) leaf sheath, (**h**) glume, (**i**) stamen, (**j**) pistil. bars: (**a**,**b**,**e**–**g**) = 1 mm; (**c**,**d**,**i**,**j**) = 500 μm; (**h**) = 2 mm.

To further characterize *OsDRP2B*, its tissue-specific expression pattern was examined using a GUS reporter driven by the *OsDRP2B* promoter. GUS staining revealed that *OsDRP2B* was expressed in the primary root tip and adventitious root tip of seedlings (Figure 4a,b). GUS signals were also detected in the maturation zones of both primary and adventitious roots, which are regions characterized by root hair differentiation and vascular tissue development (Figure 4c,d). In mature plants at the reproductive stage, GUS expression was observed in multiple aerial and reproductive tissues, including stems, leaves, leaf sheaths, glumes, stamens, and pistils (Figure 4e–j). Together, these results indicate that *OsDRP2B* is broadly expressed in rice during both vegetative and reproductive development.

### 2.3. Analysis of Agronomic Traits of Osdrp2b

**Figure 5:**
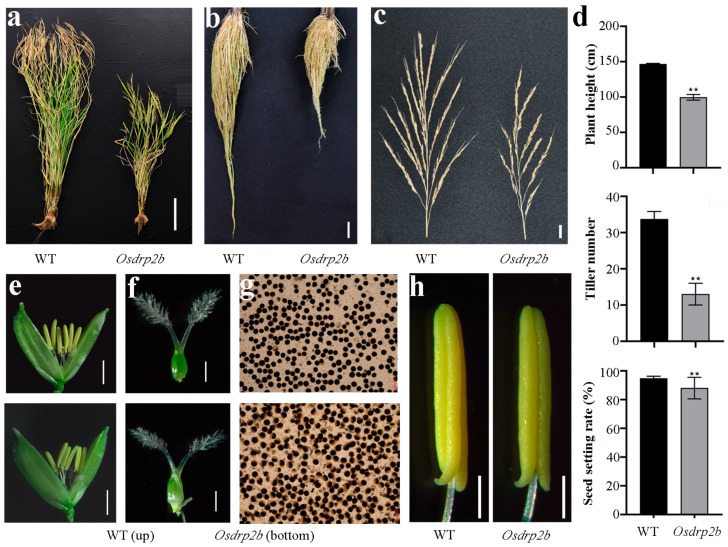
Agronomic traits and reproductive organ phenotypes of the *Osdrp2b* mutant. (**a**) Mature WT and *Osdrp2b* plants grown under field conditions. Scale bar = 20 cm. (**b**) Roots of mature WT and *Osdrp2b*. Scale bar = 2 cm. (**c**) Spikes of WT and *Osdrp2b* from field planting. Scale bar = 2 cm. (**d**) Agronomic traits at the maturation stage, including plant height, effective tiller number, stem thickness, and seed setting rate of WT and *Osdrp2b*. Values are means ± SE. Statistical significance was determined using Student’s *t*-test, and asterisks (**) indicate a significant difference from WT (*p* < 0.01). (**e**) Florets of WT (top) and *Osdrp2b* (bottom). Scale bar = 1 mm. (**f**) Pistils of WT (top) and *Osdrp2b* (bottom). Scale bar = 500 μm. (**g**) I_2_-KI staining of pollen from WT (top) and *Osdrp2b* (bottom). (**h**) Stamens of WT and *Osdrp2b*. Scale bars = 100 μm.

Due to the significant impact of root systems on shoot morphology and rice yield, we analyzed the effects of the *Osdrp2b* mutation on agronomic traits. As shown in Figure 5a, the mutant plants grown under field conditions exhibited a significantly reduced plant height compared to WT. Similarly, both root length and panicle length in mature *Osdrp2b* mutants were notably shorter than those in WT (Figure 5b,c). In addition, the mutant displayed a significantly lower seed-setting rate and reduced tiller number than WT (Figure 5d). These findings suggest that *OsDRP2B* plays a role in regulating rice yield.

To further assess the effects of the mutation at the heading stage, we examined the florets and found no significant morphological differences between the *Osdrp2b* mutant and the WT (Figure 5e). Pollen viability analysis using I_2_-KI staining showed that most mutant pollen grains were spherical but exhibited weaker staining and appeared largely unfilled compared to WT (Figure 5g). In contrast, no obvious morphological differences were observed in the stamens (Figure 5h). These results suggest that the reduced seed-setting rate in *Osdrp2b* may be attributed to decreased pollen viability.

### 2.4. Analysis of ROS Levels and Their Impact on Root Growth in the Osdrp2b Mutant

**Figure 6:**
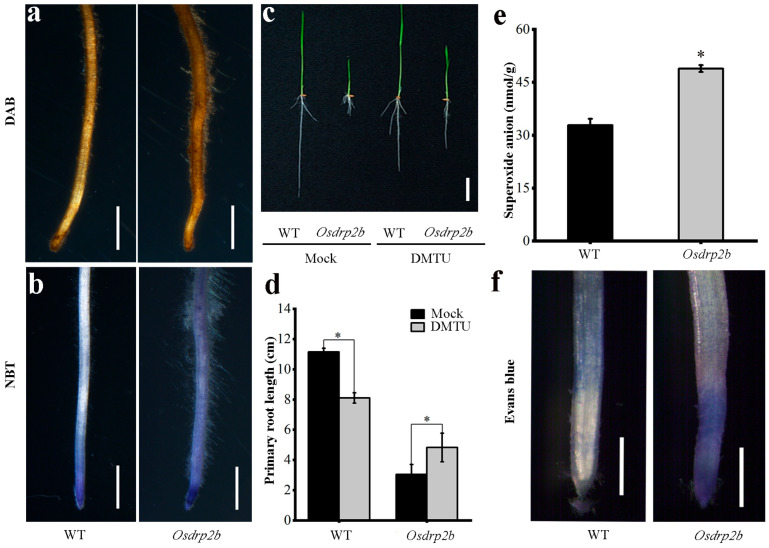
ROS analysis of the primary root in the *Osdrp2b* mutant. (**a**) DAB staining of the primary root tip of *Osdrp2b* and wild-type (WT) plants. Bars = 100 μm. (**b**) NBT staining of the primary root tip of *Osdrp2b* and WT plants. Bars = 100 μm. (**c**) Primary root length of *Osdrp2b* and WT after DMTU treatment. Bars = 2 cm. (**d**) Quantification of primary root length in *Osdrp2b* and WT after DMTU treatment in (**c**). Values are means ± SE. Statistical significance was determined using Student’s *t*-test, and asterisks (*) indicate a significant difference from WT (*p* < 0.05). (**e**) Measurement of endogenous superoxide anion content in the primary roots of *Osdrp2b* and WT. Values are means ± SE. Statistical significance was determined using Student’s *t*-test, and asterisks (*) indicate a significant difference from WT (*p* < 0.05). (**f**) Evans Blue staining of the primary root tip of *Osdrp2b* and WT plants. Bars = 1 mm.

Given the important role of ROS in regulating plant growth, we assessed ROS levels in *Osdrp2b*. DAB staining revealed a markedly deeper brown coloration in primary roots of the mutant compared with WT, indicating increased hydrogen peroxide accumulation (Figure 6a). Similarly, NBT staining showed much stronger signals in the mutant root tips (Figure 6b), suggesting elevated superoxide anion levels.

To examine the relationship between the short-root phenotype and ROS levels in *Osdrp2b*, we treated the rice plants with the ROS inhibitor DMTU. After DMTU treatment, the primary root length of the WT decreased, whereas that of the *Osdrp2b* mutant increased, nearly restoring WT levels (Figure 6c,d). This result suggests that the excessive accumulation of ROS in *Osdrp2b* inhibits root elongation. Consistently, quantification of endogenous superoxide anion content confirmed significantly higher levels in *Osdrp2b* compared with WT (Figure 6e).

To further assess the impact of ROS on cell viability, Evans Blue staining was performed. Mutant root tips accumulated significantly more dye than WT, indicating increased cell death (Figure 6f). Together, these results suggest that the elevated ROS levels in the mutant impair root growth.

### 2.5. Transcriptome Analysis of Osdrp2b

**Figure 7:**
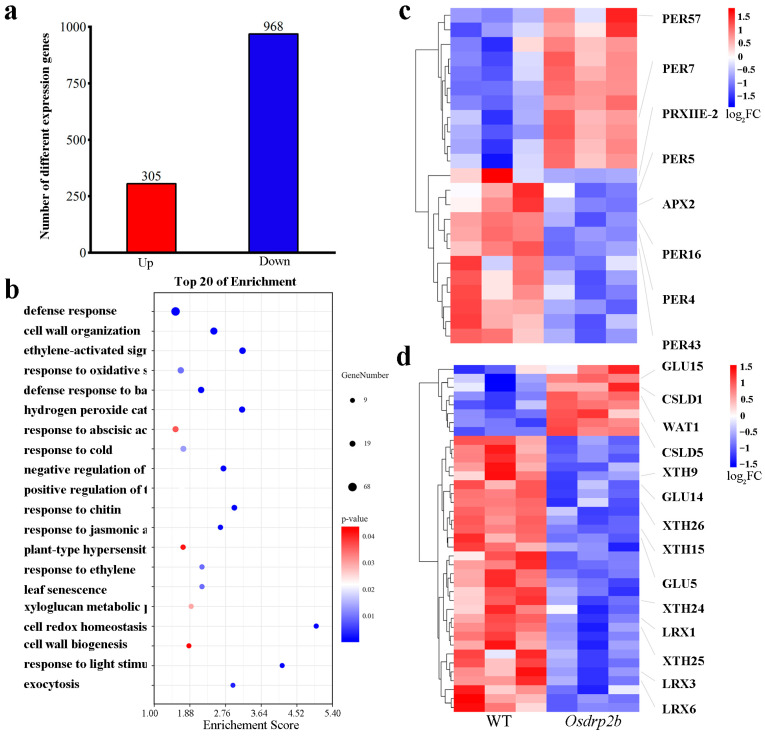
Transcriptome comparative analysis of WT and *Osdrp2b* mutant. (**a**) Number of differentially expressed genes (DEGs) that were up-regulated or down-regulated in WT versus *Osdrp2b* as determined by RNA-seq analysis. DEGs were identified with a q-value < 0.05 and a fold change > 2. (**b**) The Top20 GO terms in BP category by Gene Ontology (GO) functional enrichment analysis of differentially expressed genes (DEGs) in *Osdrp2b* vs. WT. The size of each circle corresponds to the number of genes in each pathway, while the color gradient represents the *p*-value for the enrichment of each gene set. (**c**) Heatmap showing expression profiles of DEGs involved in hydrogen peroxide catabolic process. (**d**) Heatmap showing expression profiles of DEGs involved in cell wall biogenesis and organization.

We conducted transcriptome sequencing on both WT and *Osdrp2b* plants to elucidate the molecular basis of the shortened root phenotype in *Osdrp2b*. As shown in Figure 7a, compared to the WT, 305 genes were upregulated, and 968 genes were downregulated in *Osdrp2b* (q < 0.05, FC > 2). Further enrichment analysis of the differentially expressed genes (DEGs) identified 106 significantly enriched GO terms (Table S2). In the biological process (BP) category, DEGs were primarily enriched in processes such as the hydrogen peroxide catabolic process, response to oxidative stress, xyloglucan metabolic process, and cell wall biogenesis. In the cellular components (CC) category, significant enrichment was observed in the cell wall, apoplast, and plasma membrane. Additionally, in molecular functions (MF), the DEGs were enriched in peroxidase activity, calcium ion binding, and electron transfer activity. These results suggest that the *Osdrp2b* phenotype may be associated with dysregulated expression of genes involved in these GO terms.

We ranked the GO terms within the BP category by *p*-value, as shown in Figure 7b. Among the top 20 enriched terms, processes related to ROS, such as hydrogen peroxide catabolic process and response to oxidative stress, were particularly prominent, indicating a potential role of ROS homeostasis in the *Osdrp2b* mutant. Furthermore, GO terms related to cell wall organization, cell wall biogenesis, and xyloglucan metabolic process were also enriched, indicating a potential involvement of *OsDRP2B* in cell wall structure, which is essential for cell division and elongation. We further analyzed DEGs involved in ROS metabolism and cell wall remodeling. As shown in Figure 7c, the expression of several peroxidase genes was significantly altered in the *Osdrp2b* mutant. Specifically, *PER7* and *PER57* were significantly upregulated. In contrast, *PER5*, *PER4*, *PER16*, *PER43*, *PRXIIE*, and *APX2* were significantly downregulated (fold change > 2, q < 0.05). These results suggest a potential dysregulation of hydrogen peroxide metabolism in the *Osdrp2b* mutant. Regarding cell wall biogenesis and organization, *GLU15*, *CSLD1*, and *WAT1* were upregulated in the *Osdrp2b* mutant, while *XTH9*, *XTH15*, *XTH24*, *XTH25*, *XTH26*, *LRX1*, *LRX3*, *LRX6*, *CSLD5*, *GLU5*, and *GLU14* were downregulated (Figure 7d). These differential expression patterns suggest that *Osdrp2b* disrupts ROS homeostasis and alters the balance of cell wall biosynthesis and remodeling processes, potentially contributing to its defective root phenotype.

To validate the RNA-seq results, qRT-PCR was performed on nine representative genes: *OsPER57*, *OsPER7*, *OsPER43*, *OsPER2* (ROS homeostasis), *OsXTH15*, *OsLRX6*, *OsXTH26*, *OsPGL32* (cell wall remodeling), and *OsCYCB1-2* (cell division). Except for *OsCYCB1-2*, which showed downregulation in the mutant contrary to RNA-seq results, qPCR results for the other eight genes were consistent with transcriptome trends (Supplementary Figure S2), supporting the reliability of the sequencing results.

GO enrichment also revealed hormone-related processes involving auxin, abscisic acid (ABA), ethylene, and jasmonic acid (JA) signaling (list hits > 3) (Supplementary Figure S3a). However, exogenous treatments with these hormones failed to rescue the short-root phenotype (Supplementary Figure S3b), suggesting that hormone-related transcriptional changes alone may not fully account for the observed phenotype.

To complement the conventional GO enrichment analysis, we performed Gene Set Enrichment Analysis (GSEA) based on the full gene expression dataset. A total of 124 gene sets were significantly enriched (|NES| > 1, *p* < 0.05, FDR < 0.25) (Table S5). Notably, gene sets related to the response to reactive oxygen species (NES = −2.01, FDR = 0.001), cell wall organization (NES = −1.55, FDR = 0.128), and positive regulation of the cell cycle (NES = −1.48, FDR = 0.192) were suppressed in the *Osdrp2b* mutant (Supplementary Figure S4). To examine whether these transcriptomic alterations were reflected at the physiological and biochemical levels, we analyzed ROS-related enzyme activities and cell wall components in rice roots. Under control conditions, catalase (CAT) activity was higher and peroxidase (POD) activity was lower in the *Osdrp2b* mutant than in the WT. After DMTU treatment, CAT activity decreased in the mutant, whereas POD activity increased in both genotypes (Supplementary Figure S5a,b). In addition, cell wall composition was altered in *Osdrp2b*, with reduced cellulose and increased hemicellulose contents (Supplementary Figure S5c,d). These findings indicate global dysregulation of ROS metabolism, cell wall remodeling, and cell cycle progression, consistent with the observed cellular and developmental defects in root growth.

## 3. Discussion

ROS homeostasis is essential for root development. Our findings support a model in which OsDRP2B may regulate rice root growth primarily through maintaining ROS homeostasis.

### 3.1. ROS Homeostasis Regulates Root Growth

ROS play a crucial role in plant root growth and development by modulating processes such as cell division, expansion, hormonal signaling pathways, and cell wall remodeling [7,24]. Impaired root tip growth in *Osdrp2b* is associated with disturbed ROS homeostasis, suggesting that redox imbalance contributes to the short-root phenotype (Figure 2 and Figure 6). Similar observations have been reported in *A. thaliana.* Knockdown of *SYNTAXIN OF PLANTS81* (*AtSYP81*) resulted in short roots with a severe reduction in root meristem activity and altered ROS levels [25]. Restoration of ROS levels in *Atsyp81* reversed these root growth defects. In contrast, *Osdrp2b* root growth was partially restored upon treatment with a ROS inhibitor (Figure 6d), suggesting that both excessive and insufficient ROS may impact root development. Likewise, Arabidopsis *scr* mutants also exhibit short roots and reduced epidermal cell length, which could be partially rescued by modifying redox homeostasis via *UPB1* mutation [26]. These findings align with the observed defects in cell elongation in *Osdrp2b*. Taken together, these observations reinforce the concept that maintaining ROS homeostasis, rather than a fixed ROS level, is essential for proper rice root development, although other pathways may also contribute. Future studies could explore the impact of the *UPB1* mutation in the *Osdrp2b* background to further elucidate how ROS homeostasis regulates rice root development.

ROS homeostasis is controlled by the balance between ROS production and scavenging, involving various enzymes, such as respiratory burst oxidase homologs (RBOHs), peroxidases (PER), and catalase (CAT) [7]. In *Osdrp2b* mutants, the expression of PERs was significantly affected, with most being downregulated (Figure 7c), reminiscent of the *UPB1-PER* regulatory network in Arabidopsis [27]. In a previous study, transcriptomic analyses of the elongation zone in Arabidopsis roots also revealed coordinated regulation of peroxidases and xyloglucan endo-transglycosylase/hydrolase (XTH) genes, essential for cell wall remodeling. Silencing *XTH* genes resulted in shorter root lengths [28], similarly to suppressed expression of *XTH* genes in *Osdrp2b* (Figure 7d). Consistent with these transcriptomic observations, the activities of key ROS-related enzymes were altered in *Osdrp2b* roots and partially normalized following DMTU treatment. Under control conditions, CAT activity was elevated while POD activity was reduced in the mutant, likely reflecting a compensatory response to excessive ROS accumulation. After DMTU treatment, CAT activity decreased and POD activity increased in the mutant, suggesting an improved cellular redox environment. These biochemical changes, together with the altered cell wall composition observed in *Osdrp2b* (Supplementary Figure S5c,d), provide further support that disruption of ROS homeostasis contributes to the short-root phenotype.

Transcriptome analysis revealed that multiple hormone-related signaling pathways were affected in *Osdrp2b*. However, exogenous hormone treatments failed to rescue the short-root phenotype, suggesting that the defect is not due to a simple hormone deficiency and may involve disrupted crosstalk between hormone and ROS signaling. Exogenous phytohormones, such as auxin and ABA, can induce ROS production to mediate their effects on root growth [9,29]. In *Osdrp2b*, excessive ROS accumulation may mask or override hormone effects and influence feedback regulation of hormone-related genes. Consistently, treatment with the ROS inhibitor DMTU partially restored root growth, indicating that the abnormal expression of hormone-related genes likely reflects disturbed ROS homeostasis. The precise molecular mechanisms underlying the interaction between *OsDRP2B*-mediated ROS homeostasis and hormone signaling remain to be elucidated.

### 3.2. The Conserved Role of DRP2 in Plant Development

In Arabidopsis, the *DRP* family participates in endocytosis, plasma membrane formation, pollen grain development, and cytoskeleton organization [17,30]. The *adl1A* mutant, encoding a dynamin-like protein, exhibits severely stunted roots and reduced seed set [30], phenotypes reminiscent of *Osdrp2b* (Figure 1a and Figure 5d). Furthermore, *AtDRP2*, the homolog of *OsDRP2B*, affects gametophyte development [31]. This is also consistent with our findings, where the pollen viability was reduced (Figure 5g). These parallels suggest that the *DRP2* genes may play a conserved role in regulating both root and reproductive development.

Although DRP family proteins share conserved molecular features, accumulating evidence indicates functional diversification among different DRP members in rice. For example, OsDRP1C has been shown to regulate lateral root diameter and the formation of L-type lateral roots by modulating auxin distribution through clathrin-mediated endocytosis [23]. *DBC1*, an allelic gene of *OsDRP2B*, primarily controls plant height by regulating cell division [21]. Moreover, BC3/OsDRP2B was previously reported to participate in membrane trafficking and endocytosis, thereby affecting the plasma membrane abundance of cellulose synthase and secondary wall structure [20]. Building on these studies, our work suggests that OsDRP2B is also involved in rice root development, contributing to root growth, at least in part, through the regulation of ROS homeostasis. This observation expands the current understanding of the functional diversity of DRP family members.

DRPs are involved in cell plate formation via clathrin-mediated endocytosis, influencing cytokinesis [32]. For instance, the *Atdrp1* mutant exhibited defects in cell plate assembly, ultimately leading to abnormal cell division, and showed hypersensitivity to the membrane trafficking inhibitor, resulting in restricted root elongation [33]. These findings align with our results that *OsDRP2B* affects cell division.

Based on studies in Arabidopsis and other rice DRP homologs, we speculate that OsDRP2B may be involved in regulating ROS homeostasis through clathrin-mediated endocytosis. Our transcriptome analysis revealed a significant enrichment of endocytosis-related genes among the differentially expressed genes in the *Osdrp2b* mutant (Figure 7b), suggesting a possible link between OsDRP2B and endocytosis. However, direct experimental evidence is still lacking. In Arabidopsis, DRP2-mediated clathrin-dependent endocytosis is critical for the spatial regulation of RBOHD, affecting ROS production [19,34,35]. Similarly, in rice, the *Osdrp1c* mutant exhibits reduced endocytosis and altered auxin transporter localization [23], suggesting a conserved role of DRPs in vesicle trafficking. Furthermore, *OsDRP2B* has been previously implicated in membrane trafficking and cellulose biosynthesis by regulating the plasma membrane abundance of OsCESA4 [20]. The involvement of OsDRP2B in ROS regulation via endocytosis remains hypothetical and requires future experimental validation, such as endocytic activity assays or subcellular localization of ROS-related proteins.

**Figure 8:**
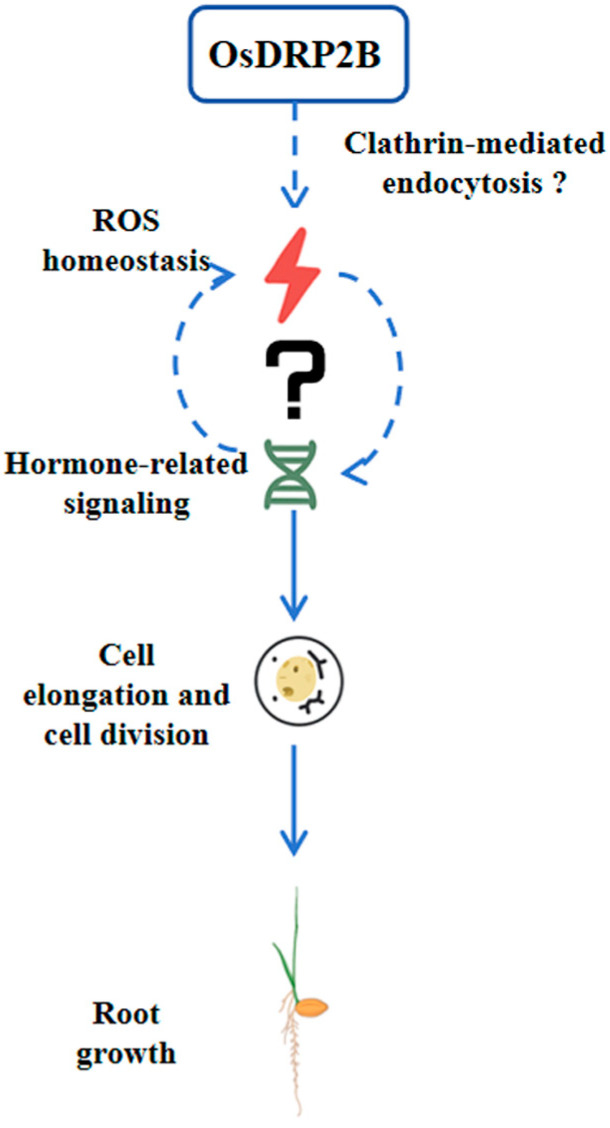
A proposed working model of OsDRP2B in regulating rice root growth. OsDRP2B is proposed to modulate ROS homeostasis, possibly via clathrin-mediated endocytosis. Altered ROS levels may directly affect cell division and elongation in the root tip and/or indirectly influence these processes through crosstalk with hormone signaling pathways. Through these mechanisms, OsDRP2B ultimately regulates rice root growth.

In conclusion, OsDRP2B is a key regulator of rice root development, as the *Osdrp2b* mutant exhibits short roots due to reduced cell elongation and impaired cell division in the root tip. Elevated ROS levels in the mutant and restoration of root growth by ROS inhibitors indicate that OsDRP2B contributes to root development via maintaining ROS homeostasis. Mechanistically, OsDRP2B may act via clathrin-mediated endocytosis to modulate ROS balance, which in turn interacts with hormone signaling to regulate cell division and elongation, ultimately controlling root growth (Figure 8). These findings provide a conceptual framework for the working model of OsDRP2B and highlight its potential as a target for optimizing rice root architecture and improving yield.

## 4. Materials and Methods

### 4.1. Plant Materials and Growth Conditions

The *Osdrp2b* mutant was isolated from an ethyl methane sulfonate (EMS)-mutagenized rice (*Oryza sativa* L. *India* cv. Kasalath) mutant library. Wild-type (WT) plants of the same genetic background (Kasalath) were used as controls in all experiments.

Seeds were surface-sterilized and germinated in distilled water for 2 days, after which uniform seedlings were transferred to a greenhouse and cultivated in standard rice culture medium (pH 5.5), following the protocol described by Zhu et al. [36]. The climate chamber conditions were maintained at 32/22 °C (day/night), with 60–70% relative humidity, a light intensity of 16,000 lux, and a 12 h photoperiod. The rice culture medium was renewed weekly.

To investigate the impact of reactive oxygen species (ROS) on root growth and development in *Osdrp2b*, seedlings were cultured in a solution supplemented with 1 mM N, N′-dimethylthiourea (DMTU). To evaluate the effect of phytohormones on the root growth of *Osdrp2b*, seedlings were treated with culture solutions supplemented with 0.2 μM 2,4-D, 0.5 μM ABA, 4 mg/L ethephon, or 0.5 μM JA. Seedlings grown in hormone-free culture solution were used as controls. All treatments were performed for 7 days unless otherwise indicated. At least three independent biological replicates were conducted for each treatment.

### 4.2. Mapping and Cloning of the OsDRP2B

To map the *OsDRP2B* locus, the *Osdrp2b* homozygous mutant was crossed with Nipponbare, a *japonica* rice wild-type to generate an F_1_ mapping population. From the F_2_ generation derived by selfing the F_1_, 30 and 519 short-root lines exhibiting a phenotype consistent with *Osdrp2b* were selected for coarse and fine mapping of *OsDRP2B*, respectively. Genomic DNA was extracted from seedlings using a standard CTAB method. PCR-based InDel markers polymorphic between Kasalath and Nipponbare were developed and used for linkage analysis. The primers used for mapping are listed in Supplementary Data Table S1.

*OsDRP2B* was mapped to a 109 kb region on chromosome 2, between the sequence marker sites In1 and In4. Candidate genes within this interval were identified based on the Rice Genome Annotation Project database. Coding sequences of candidate genes were amplified and sequenced to identify potential EMS-induced mutations.

### 4.3. Vector Construction and Plant Transformation

For genetic complementation, the full-length coding sequence of *OsDRP2B* was amplified from wild-type rice cDNA and cloned into the binary vector *pCAMBIA1300* under the control of the CaMV 35S promoter. For tissue expression patterns analysis, a 2456 bp *OsDRP2B* promoter was amplified by PCR and cloned into *pCAMBIA11300NHGUS*. All constructs were confirmed by sequencing. The resulting vectors were introduced into wild-type or mutant rice via *Agrobacterium tumefaciens*-mediated transformation. Transgenic seedlings were selected on hygromycin-containing medium and positive plants were further confirmed by PCR amplification of the hygromycin resistance (hpt) gene. The primers used for genotyping are listed in Supplementary Data Table S1.

### 4.4. Histochemical Analysis and GUS Assay

Histochemical GUS analysis was performed following the protocol described by Ding et al. [37]. Transgenic plant samples at the indicated developmental stages were incubated in GUS staining solution (100 mM NaH_2_PO_4_ buffer pH 7.0, 0.5% Triton X-100, 0.5 mg mL^−1^ X-Gluc and 20% methanol). Samples were vacuum-infiltrated for 1 min and then incubated overnight at 37 °C. After staining, tissues were destained with 70% ethanol, then were mounted on slides and photographed using a stereo microscope (Leica MZ95, Nussloch, Germany).

### 4.5. Acetocarmine and EdU Staining

Acetocarmine and EdU staining were performed as previously described by Ding et al. [37]. Briefly, for acetocarmine staining, root tips of 7-day-old WT and *Osdrp2b* seedlings were stained with 1% acetocarmine for 10 min in the dark, washed with 45% acetic acid, and observed under a stereo microscope (Leica MZ95). For EdU staining, roots of 4-day-old WT and *Osdrp2b* seedlings were incubated in 20 μM EdU medium for 2 h, fixed for 30 min, and washed three times with phosphate buffer (pH 7.2). Samples were then incubated in EdU detection solution for 30 min in the dark and imaged using a confocal microscope (Zeiss LSM 510, Jena, Germany).

### 4.6. Histological Observation

Histological observation was conducted as previously described by Ye et al. [38]. Root tips from 3-day-old rice seedlings were excised and fixed overnight at 4 °C in 0.1 M sodium phosphate buffer (pH 7.2) containing 2.5% glutaraldehyde. Samples were washed three times for 30 min each in the same buffer and then post-fixed in 1% osmium tetroxide (OsO_4_) for 4 h at room temperature, followed by a 30 min wash in the same buffer. Samples were dehydrated through a graded ethanol series and embedded in pure Spurr resin, which was polymerized overnight at 70 °C. Semithin sections (2 μm thick) were cut with a diamond knife on a Power Tome XL microtome (RMC-Boeckeler Instruments, Tucson, AZ, USA) and stained with 0.1% methylene blue for 3–5 min at 70 °C. After rinsing with distilled water, images were captured using a Nikon 90i microscope (Nikon, Tokyo, Japan). At least three independent biological replicates were analyzed.

### 4.7. Detection of Reactive Oxygen Species (ROS), Antioxidant Enzyme Activities and Cell Death

Hydrogen peroxide (H_2_O_2_) accumulation in rice roots was detected using an endogenous peroxidase-dependent staining method with 3,3′-diaminobenzidine (DAB; Sigma-Aldrich, St. Louis, MO, USA). Roots were incubated in 1 mg/mL DAB prepared in double-distilled water for 20 min at room temperature (22–25 °C) in the dark. Superoxide (O_2_^−^) production was examined by staining root tissues with 2 mM nitroblue tetrazolium chloride (NBT; Sigma-Aldrich) in 10 mM phosphate buffer (pH 7.0) for 10 min at room temperature under ambient light. Cell death was assessed using 1 mg/mL Evans Blue (Sigma-Aldrich) in double-distilled water for 10 min at room temperature, followed by three washes with distilled water to remove excess dye. Stained tissues were mounted on glass slides and imaged using a Nikon 90i microscope (Nikon, Japan). More than ten tissue samples were examined for each genotype, and all experiments were repeated at least twice. Representative images are shown.

Quantitative measurements of superoxide anion (O_2_^−^) levels and the activities of antioxidant enzymes, peroxidase (POD) and catalase (CAT), in rice seedlings were performed using commercial kits (Suzhou Grace Biotechnology Co., Ltd., Suzhou, China), following the manufacturer’s instructions. At least three independent biological replicates were analyzed.

### 4.8. Transcriptome Analysis and qRT-PCR

Transcriptome analysis by RNA sequencing was conducted as previously described by Ma et al. [39]. Roots of 7-day-old WT and *Osdrp2b* plants, grown in solution culture were harvested for RNA-seq experiments. Three biological replicates for each sample were collected. Total RNA extraction, cDNA library construction, and sequencing were conducted by OE Biotech Co., Ltd. (Shanghai, China) using the Illumina NovaSeq 6000 platform. GSEA was performed using the GSEA software (version V2.0.1.8, www.oebiotech.com). A predefined gene set was used, and all genes were ranked based on their differential expression between the two sample types. The analysis tested whether members of the predefined gene set were significantly enriched at the top or bottom of the ranked gene list.

For qRT-PCR analysis, total RNA was extracted from 7-day-old WT and *Osdrp2b* seedlings using RNAiso plus (Takara, Kyoto, Japan) and quantified by NanoDrop (Thermo Fisher Scientific, Waltham, MA, USA). cDNA synthesis was performed with the ABScript II First-Strand Synthesis Kit (ABclonal, Wuhan, China). qRT-PCR was carried out on a LightCycler 96 System (Roche, Basel, Switzerland) using ChamQ SYBR qPCR Master Mix (Vazyme Biotech, Nanjing, China). Primers are listed in Table S1.

### 4.9. Pollen Activity Staining Assay

Spikelets about to flower from WT and *Osdrp2b* plants were carefully selected for examination. After gently opening the hull, anthers were placed in several drops of 1% iodine-potassium iodide (I_2_-KI; Sigma-Aldrich) solution on glass slides and gently crumbled to release pollen grains. Samples were stained at room temperature (22–25 °C) for 5 min before observation. Pollen viability was assessed under a Nikon Eclipse 90i light microscope (Nikon, Japan). More than five plants per genotype were analyzed, and the experiment was repeated at least twice. Representative images are shown.

### 4.10. Determination of Cellulose and Hemicellulose Contents

The cellulose and hemicellulose contents in the roots of 7-day-old rice seedlings were measured using commercial kits (Cellulose content determination kit, G0715W; Hemicellulose content determination kit, G0716W; Suzhou Grace Biotechnology Co., Ltd., Suzhou, China) according to the manufacturer’s instructions. At least three independent biological replicates were analyzed.

## Data Availability

The RNA-seq data generated in this study have been deposited in the NCBI BioProject database under accession number PRJNA1383587. Other data supporting the findings of this study are available from the corresponding author upon reasonable request.

## Supplementary Materials

The following supporting information can be downloaded at: https://www.mdpi.com/article/10.3390/plants15020313/s1, Figure S1: Root radial expansion analysis in the *Osdrp2b* mutant. (a) Cross sections of the maturation zone (top), elongation zone (middle), and meristematic zone (bottom) in 3-day-old WT and *Osdrp2b* roots. Scale bars = 100 μm. (b) Quantification of cortical cell width in roots of 3-day-old WT and *Osdrp2b* seedlings. Values represent means ± SE. Significant differences were determined using Student’s *t*-test (*p* < 0.05); Figure S2: Validation of RNA-seq results by qRT-PCR. Relative expression levels of nine selected genes involved in ROS homeostasis (*OsPER57*, *OsPER7*, *OsPER43*, *OsPER2*), cell wall remodeling (*OsXTH15*, *OsLRX6*, *OsXTH26*, *OsPGL32*), and cell division (*OsCYCB1–2*) in 7-day-old WT and *Osdrp2b* seedlings as determined by qRT-PCR. Gene expression levels were normalized to *OsACTIN1*. Values represent means ± SE. Significant differences were determined using Student’s *t*-test (*p* < 0.05); Figure S3: Hormone-related GO term enrichment and hormone treatment assays. (a) GO enrichment analysis of differentially expressed genes (DEGs) revealed significant enrichment of hormone-related biological processes. Hormone-related GO terms are ranked by the number of DEGs (list hits) associated with each term. (b) Phenotypes of WT and *Osdrp2b* seedlings grown in nutrient solution with or without 0.2 μM 2,4-D (auxin), 0.5 μM ABA, 4 mg/L ethephon (ethylene-releasing compound), and 0.5 μM JA for 7 days. WT and *Osdrp2b* seedlings grown without hormones serve as control groups. Bar = 2 cm; Figure S4: Gene Set Enrichment Analysis (GSEA) in *Osdrp2b*. GSEA was performed using the full gene expression dataset to identify significantly enriched biological processes. Representative suppressed gene sets in the *Osdrp2b* mutant are shown, including response to reactive oxygen species (a), cell wall organization (b), and positive regulation of the cell cycle (c). Significance thresholds were set at |NES| > 1, *p* < 0.05, and FDR < 0.25. Figure S5: ROS-related enzyme activities and cell wall composition in *Osdrp2b*. (a,b) Catalase (CAT) and peroxidase (POD) activities in 7-day-old WT and *Osdrp2b* seedlings under control conditions and after DMTU treatment. Different letters (a, b, c) indicate significant differences among groups (one-way ANOVA, *p* < 0.05). (c,d) Cellulose and hemicellulose contents in roots of 7-day-old WT and *Osdrp2b* seedlings. Significant differences between genotypes were assessed by Student’s *t*-test and are indicated by asterisks (* *p* < 0.05); Table S1. Primers used in the study; Table S2. Enriched GO terms in *Osdrp2b* compared WT (*p* < 0.05); Table S3. The expression of the DEGs enriched to hydrogen peroxide catabolic process (q < 0.05 and FC > 2); Table S4. The expression of the DEGs enriched to cell wall biogenesis and cell wall organization (q < 0.05 and FC > 2); Table S5. Significantly enriched gene sets identified by Gene Set Enrichment Analysis (GSEA) (|NES| > 1, *p* < 0.05, FDR < 0.25).
