# Supplementary material for: Functional Analysis of OsDRP2B in Rice Root Development
Source: Plants (Basel). 2026 Jan 21;15(2):313. doi: 10.3390/plants15020313 (PMC12845046; doi:10.3390/plants15020313)
Supplement: Supplementary file 1 [file plants-15-00313-s001.zip › highlighted version Supplementary_Figures.pdf]

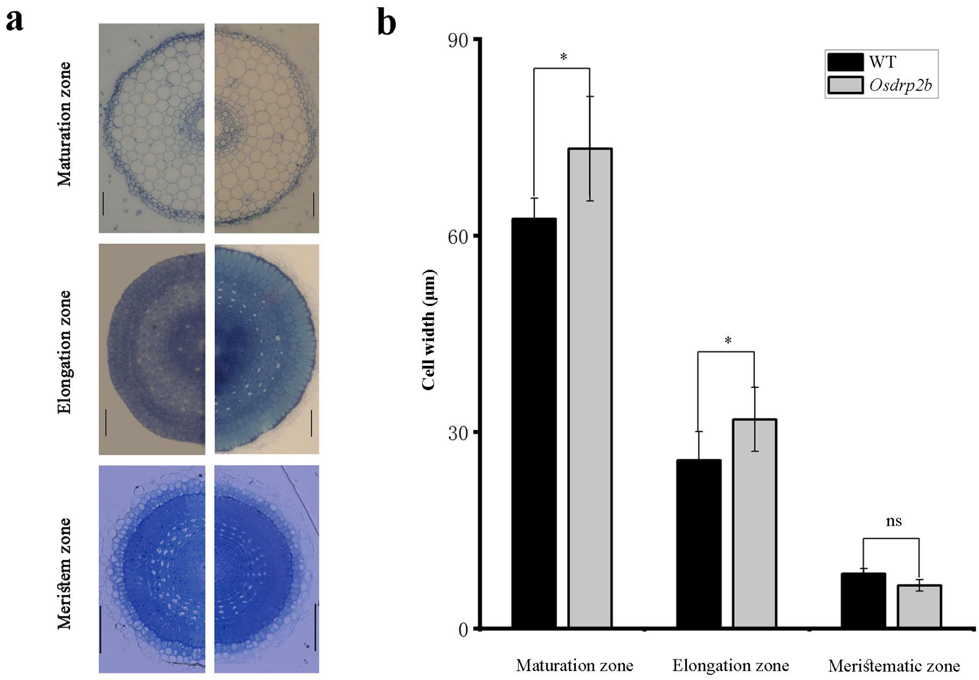

Supplementary Figure S1 Root radial expansion analysis in the *Osdrp2b* mutant. (a) Cross sections of the maturation zone (top), elongation zone (middle), and meristematic zone (bottom) in 3-day-old WT and *Osdrp2b* roots. Scale bars = 100 μm. (b) Quantification of cortical cell width in roots of 3-day-old WT and *Osdrp2b* seedlings. Values represent means ± SE. Significant differences were determined using Student's t-test ($P < 0.05$).

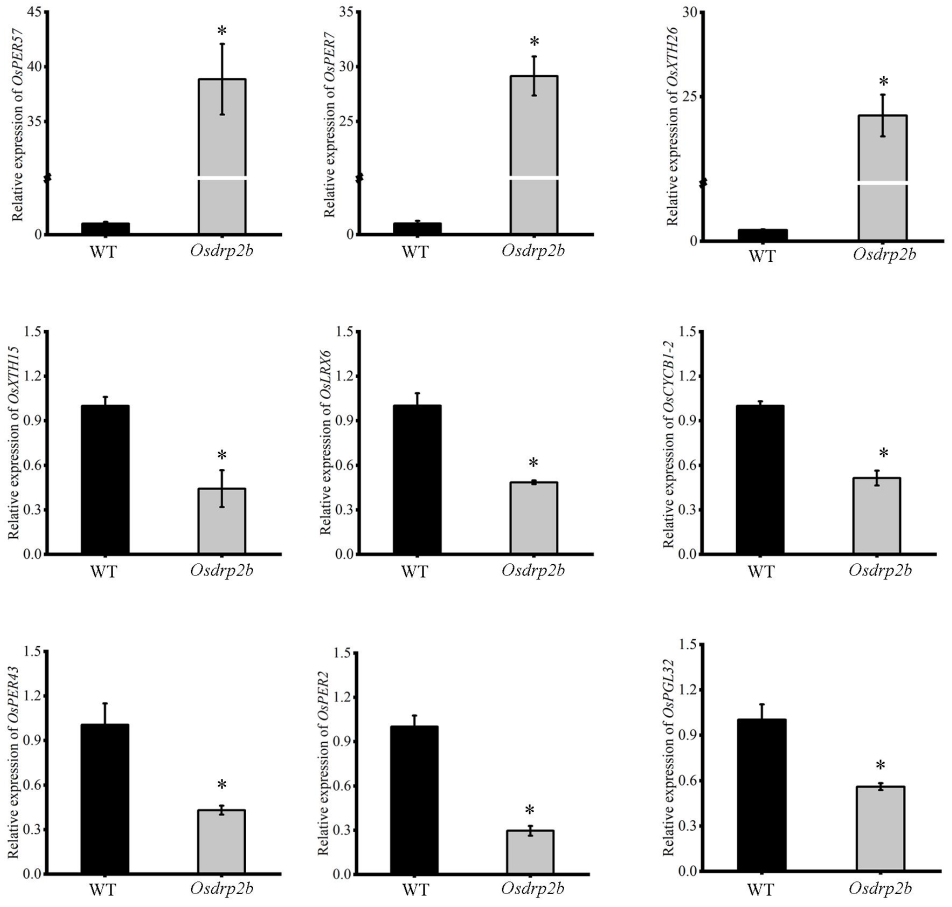

Supplementary Figure S2 Validation of RNA-seq results by qRT-PCR. Relative expression levels of nine selected genes involved in ROS homeostasis (*OsPER57*, *OsPER7*, *OsPER43*, *OsPER2*), cell wall remodeling (*OsXTH15*, *OsLRX6*, *OsXTH26*, *OsPGL32*), and cell division (*OsCYCB1-2*) in 7-day-old WT and *Osdrp2b* seedlings as determined by qRT-PCR. Gene expression levels were normalized to *OsACTIN1*. Values represent means ± SE. Significant differences were determined using Student's t-test ($P < 0.05$).

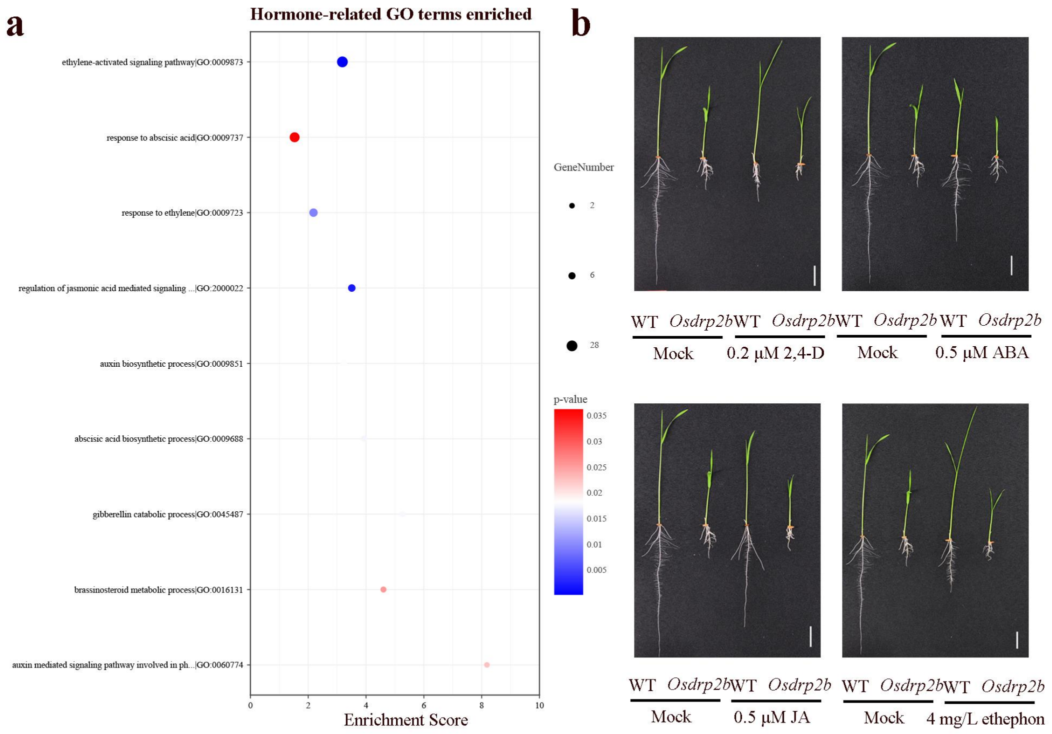

Supplementary Figure S3 Hormone-related GO term enrichment and hormone treatment assays. (a) GO enrichment analysis of differentially expressed genes (DEGs) revealed significant enrichment of hormone-related biological processes. Hormone-related GO terms are ranked by the number of DEGs (list hits) associated with each term. (b) Phenotypes of WT and *Osdrp2b* seedlings grown in nutrient solution with or without 0.2 μM 2,4-D (auxin), 0.5 μM ABA, 4 mg/L ethephon (ethylene-releasing compound), and 0.5 μM JA for 7 days. WT and *Osdrp2b* seedlings grown without hormones serve as control groups. Bar = 2 cm.

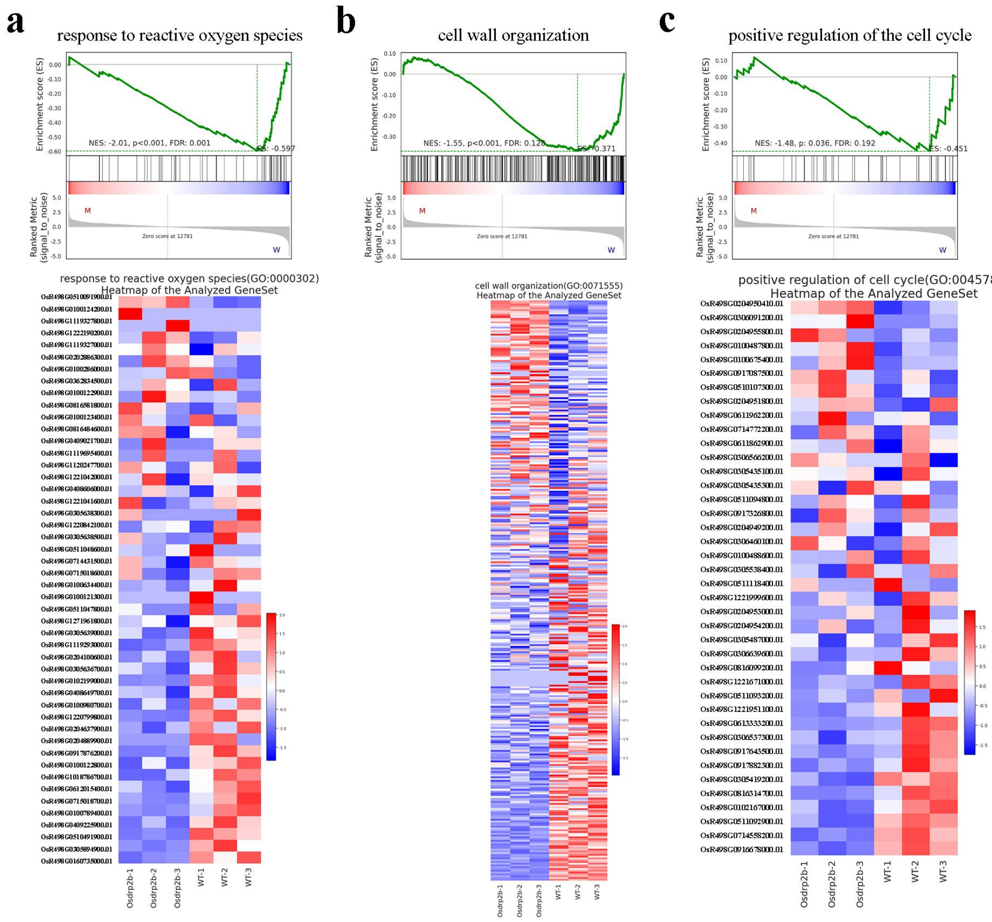

Supplementary Figure S4. Gene Set Enrichment Analysis (GSEA) in *Osdrp2b*. GSEA was performed using the full gene expression dataset to identify significantly enriched biological processes. Representative suppressed gene sets in the *Osdrp2b* mutant are shown, including response to reactive oxygen species (a), cell wall organization (b), and positive regulation of the cell cycle (c). Significance thresholds were set at |NES| > 1, $P < 0.05$, and FDR < 0.25.

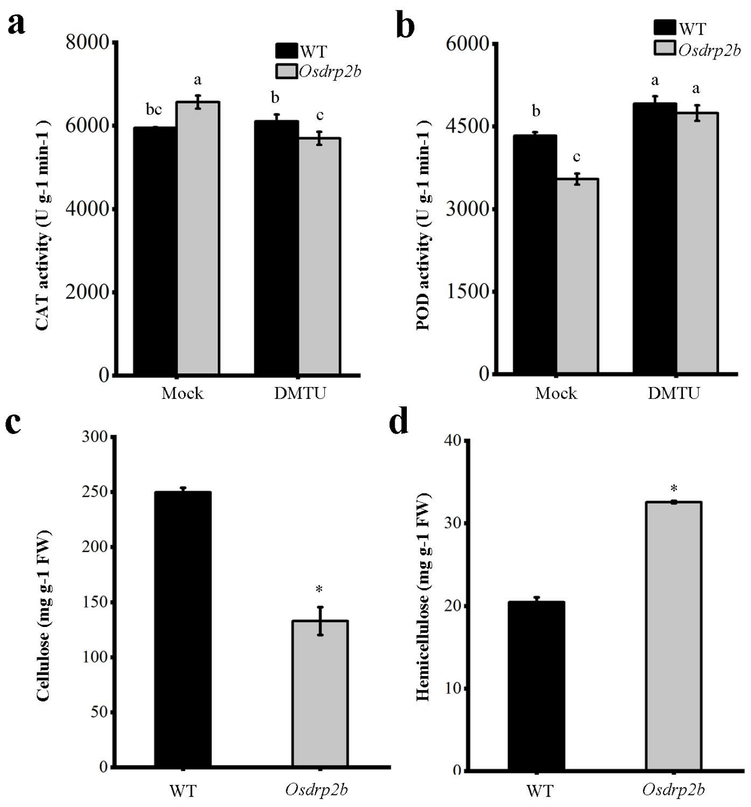

Supplementary Figure S5. ROS-related enzyme activities and cell wall composition in *Osdrp2b*. (a–b) Catalase (CAT) and peroxidase (POD) activities in 7-day-old WT and *Osdrp2b* seedlings under control conditions and after DMTU treatment. Different letters (a, b, c) indicate significant differences among groups (one-way ANOVA, $P < 0.05$). (c–d) Cellulose and hemicellulose contents in roots of 7-day-old WT and *Osdrp2b* seedlings. Significant differences between genotypes were assessed by Student's t-test and are indicated by asterisks (*$P < 0.05$).
